# Social and Non-social Reward: A Preliminary Examination of Clinical Improvement and Neural Reactivity in Adolescents Treated With Behavioral Therapy for Anxiety and Depression

**DOI:** 10.3389/fnbeh.2019.00177

**Published:** 2019-08-23

**Authors:** Karen T. G. Schwartz, Maria Kryza-Lacombe, Michael T. Liuzzi, V. Robin Weersing, Jillian Lee Wiggins

**Affiliations:** ^1^San Diego State University/University of California, San Diego Joint Doctoral Program in Clinical Psychology, San Diego, CA, United States; ^2^Department of Psychology, San Diego State University, San Diego, CA, United States

**Keywords:** reward, behavioral therapy, fMRI, adolescents, anxiety, depression

## Abstract

**Background:**

Pediatric anxiety and depression are highly prevalent and debilitating disorders that often co-occur. Neural circuitry of reward processing has been shown to be implicated in both, and there is an emerging evidence base linking treatment response to brain patterns of reward processing. The current study aimed to add to this literature by investigating the association between clinical improvement and social and non-social reward in youth previously treated for anxiety and depression.

**Methods:**

The current study leveraged clinical improvement data from a successful randomized controlled trial testing the efficacy of a transdiagnostic, brief behavioral treatment for youth diagnosed with anxiety or depression. Participants (*N* = 15) interested in engaging in a neuroimaging follow-up underwent an fMRI scan, during which they completed social (i.e., Face Task) and non-social (i.e., Piñata Task, a youth-friendly monetary incentive delay task) reward tasks. Whole-brain activation and functional connectivity analyses identified neural responses to the tasks separately; a third set of analyses directly compared clinical improvement-related findings to understand the impact of task context on neural reactivity to reward.

**Results:**

Activation-based findings were sparse; however, connectivity as a function of degree of treatment response was apparent and robust. Within the context of social reward, significant clusters within frontal and temporal regions driven by happy face contrasts, the social reward stimulus, were observed. This supports connectivity between these regions and both amygdala and ventral striatum seeds as a function of degree of clinical improvement. Connectivity within the context of non-social reward also yielded significant clusters in temporal and parietal regions. Here too, the magnitude and direction of region coupling depended on the degree of clinical improvement and the task conditions. No differences in connectivity by task type as a function of clinical improvement were found.

**Conclusion:**

Findings serve as preliminary evidence that neural regions found to be related to clinical improvement within the context of social and non-social reward are similar to regions that have been shown to support reward processing in normative samples. Implications for treatment and future work are discussed.

## Introduction

Pediatric anxiety and depression are highly prevalent, debilitating, and associated with a chronic course and long-term impairment (e.g., Merikangas et al., 2010). They frequently co-occur, both concurrently and sequentially (Garber and Weersing, 2010), and data suggest shared genetic risk (Thapar and McGuffin, 1997). Anxiety and depression also respond to the same classes of psychosocial (e.g., cognitive and behavioral therapies) and pharmacological (e.g., SSRIs) interventions (Compton et al., 2004), and treatment of one target disorder may lead to cross-over effects on the other disorder (e.g., interventions that target depression may reduce non-targeted anxiety symptoms; Garber et al., 2016).

This work has served as the rationale for the development of transdiagnostic interventions designed to target core processes across anxiety and depression and treat them as a unified problem area. In adults, unified protocols have demonstrated superior symptom improvement across clinical domains, both compared to control conditions and disorder-specific care (see McEvoy et al., 2009 for a review). The few studies that have tested transdiagnostic protocols in pediatric samples have also documented the efficacy in targeting internalizing disorders (Chu et al., 2016; Ehrenreich-May et al., 2017; Weersing et al., 2017), suggesting shared processes of disorder and recovery across anxiety and depression. However, such findings are in contrast to evidence that anxiety and depression differ in their responses to intervention. For instance, interventions targeting anxiety have the largest intervention effect sizes in the pediatric literature; in contrast, depression treatment effects are the smallest in the field (Weisz et al., 2017). Furthermore, pediatric unified protocols have evidenced better effects on anxiety, compared to depression outcomes, despite success overall (Queen et al., 2014; Weersing et al., 2017). Thus, additional work must be done to examine underlying dimensional factors that cross diagnostic boundaries and may more effectively account for observed differences in response to care in internalizing youth. Such efforts are aligned with the priorities of the National Institutes of Health (NIH) in an effort to improve the efficacy of treatments (e.g., Insel et al., 2010).

One neurobiological mechanism that has been implicated across anxiety and depression is reward processing, in both social and non-social contexts. Reward processing encompasses neural reactivity associated with anticipation and consumption of positive gains, such as monetary winnings or social approval, as well as behavioral learning that motivates future actions. Studies of reward processing in pediatric samples have included tasks based on monetary incentives or tasks focused on social appraisal. The former maps onto the adult literature, as money is an ecologically valid incentive that is easily manipulated and distributed by study personnel. Social reward is a relatively new area of interest relevant to pediatric samples, given the developmental alterations in social valuation during adolescence (Steinberg and Morris, 2001). Indeed, adolescence is a period of substantial neural maturation in areas associated with reward (e.g., Ernst et al., 2006; Galvan, 2010). Concurrent developmental changes include enhanced need for social inclusion and peer acceptance (e.g., Choudhury et al., 2006). Thus, happy faces may be particularly rewarding during this developmental period (Scherf et al., 2012). Furthermore, happy faces are utilized to signal success on achievement-oriented tasks, such as academic assignments; therefore, happy faces are emotionally salient and socially relevant cues that can be reasonably expected to probe reward processing neural circuitry in youth.

Anhedonia, or the motivation and ability to seek and experience rewarding activities, is a core diagnostic feature of depression, though not anxiety. Anhedonia has been shown to serve as a phenotype of aberrant integration of reward and arousal, above and beyond other symptoms of internalizing disorders in youth (Pornpattananangkul et al., 2019). Generally, youth with or at risk for depression evidence blunted patterns of response in areas associated with reward, and signals appear to be further diminished by intensified cognitive control (e.g., Forbes et al., 2006, 2009; Chantiluke et al., 2012; Wiggins et al., 2017). Four treatment trials targeting depression in youth included task-based neuroimaging components prior to treatment, only (i.e., baseline; Forbes et al., 2010), or at both pre- and post-treatment timepoints (i.e., baseline and follow-up; Straub et al., 2015; Chuang et al., 2016; Mori et al., 2016); three studies probed non-social (i.e., monetary) reward processing (Forbes et al., 2010; Straub et al., 2015; Mori et al., 2016). Pre- to post-treatment changes in brain patterns suggested that aberrant responses to reward conditions “normalized” in youth as a function of treatment (i.e., mirrored patterns observed in healthy controls; Mori et al., 2016). Pre-post signal reductions in areas associated with emotion regulation were observed as a function of treatment engagement and related significantly to larger depression symptom reductions at post and follow-up (Straub et al., 2015).

The conceptual connection between anhedonia, depression and reward processing is clear; however, reward processing has also be implicated in anxiety in a different fashion. Anxiety disorders are characterized by avoidance of anxiety-provoking stimuli, escape behaviors when exposed to anxiety triggers, and negative “reward” of avoidance and escape through the reduction of anxious distress. In adolescence, avoidance of social interactions becomes particularly prevalent due to intensified concerns regarding peer approval and acceptance. Though smaller than the depression literature, neuroimaging findings on reward processing in pediatric anxiety have begun to accrue. fMRI studies enrolling anxious vs. non-anxious youth evidenced increased striatal, frontal, and limbic reactivity during social and non-social reward tasks (e.g., Guyer et al., 2012; Benson et al., 2015; Jarcho et al., 2015); of note, these studies did not include a treatment component. To date, five reports citing data from four independent trials serve as the current literature base on neural predictors of response to psychosocial interventions targeting pediatric anxiety (McClure et al., 2007; Maslowsky et al., 2010; Kujawa et al., 2016; Burkhouse et al., 2017; White et al., 2017). Three studies incorporated fMRI data from baseline and follow-up (McClure et al., 2007; Maslowsky et al., 2010; White et al., 2017), while two representing the same trial utilized baseline data only (Kujawa et al., 2016; Burkhouse et al., 2017). However, no investigations examined reward processing, specifically, as a neural predictor or mechanism of treatment response in this population.

It is notable that none of the published findings tested a transdiagnostic protocol, yet the majority of samples evidenced substantial diagnostic comorbidity. Furthermore, changes in striatal reactivity in response to a depression-focused intervention was associated with cross-over effects, such that changes of greater magnitude predicted a faster rate of decline in anxiety symptoms across time (Forbes et al., 2010). Thus, work within the transdiagnostic realm is warranted. Moreover, given the relevance of social processing in anxiety and depression, disorders frequently characterized by interpersonal difficulties, focusing on social in addition to non-social reward is necessary.

In sum, the literature base on neural mechanisms of treatment response in internalizing youth is in its infancy. Studies are few, segregated by diagnosis, and lack replication within treatment modality across independent teams. Additionally, treatment paradigms employed were diagnosis-specific, despite high rates of comorbidity within samples, and typically focused on only one aspect of reward (i.e., non-social reward). To address these gaps in the literature, the current study leveraged resources from a successful randomized controlled trial (RCT) to examine neural mechanisms within the context of a treatment trial in a comorbid sample of anxious-depressed youth. Specifically, we sought to examine both social and non-social reward processing as promising neural mechanisms of clinical improvement in a subsample of youth, ages 8−16 years, enrolled in a multi-site RCT investigating the effectiveness of a transdiagnostic brief behavioral therapy (BBT) for pediatric anxiety and depression (Weersing et al., 2017). BBT may be a particularly relevant treatment paradigm to evaluate the relationship between both social and non-social reward processing and treatment response due to the behavioral target of intervention. That is, both anxiety and depression are characterized by avoidance of negative affect and behavioral withdrawal, including from social situations. Behavioral interventions directly target avoidance by increasing reinforcement in response to engagement and decreasing reinforcement for avoidance behaviors. Furthermore, behavioral interventions are developmentally appropriate for youth, as behavioral tasks are active, concrete, and cognitively straightforward (Martin and Oliver, 2018). So, we re-contacted this sample to collect neuroimaging data post-treatment with the aim of relating imaging data to variables defined during the original RCT participation, such as baseline characteristics of youth and BBT treatment response.

The original BBT trial evidenced statistically significant positive effects across measures. Youth who received BBT were more likely to be categorized as treatment responders by the post-treatment assessment [i.e., Clinical Global Impressions, Improvement Scale (CGI-I; Guy, 1976) ≤2] and evidenced improved functioning compared to those receiving assisted referral to care (ARC; control condition). Furthermore, the rate of functional improvement among those who received BBT was significantly faster than improvements reported by those in the ARC condition (see Weersing et al., 2017 for full methods and CONSORT). The BBT intervention targeted avoidance across anxiety and depression by promoting graded engagement in important life tasks, providing participants with concrete, alternate experiences meant to be rewarding (Weersing et al., 2008). Some of the targeted tasks were social in nature while others were based on success experiences, as youth have a number of difficulties with achievement-oriented activities. We thus took the opportunity to probe the distinction between social (i.e., happy face) and non-social (i.e., monetary) reward tasks, as a comparison like this in the same sample has yet to be published. Additionally, the developmental maturation of reward processing circuitry in adolescence suggests differences in salience and associated neural reactivity in response to social and monetary reward cues can be expected.

To our knowledge, this study is the first to (a) evaluate social reward in internalizing youth within the context of treatment (b) inform the relationship between reward processing and clinical improvement in response to a youth-focused transdiagnostic psychosocial intervention, and (c) analyze data from one sample of youth who each performed two tasks. Planned statistical analyses represent secondary analyses of data from a completed clinical trial (Weersing et al., 2017), combined with original neuroimaging data collection. Scans were performed post-treatment to generate hypotheses regarding the long-term role of treatment response in reward processing. These efforts are exploratory in nature to contribute to the establishment of a literature base of neural mechanisms of treatment response in internalizing youth and bolster future research.

## Materials and Methods

The protocol for the neuroimaging follow-up was reviewed and approved by the University of California, San Diego Institutional Review Board. Secondary approval was obtained from San Diego State University’s Institutional Review Board. Written informed consent was obtained from all participating caregivers and adolescents over 18 years of age, prior to the administration of any study materials, in accordance with the Declaration of Helsinki. Adolescents younger than 18 provided assent, in addition to their caregiver’s written informed consent to participate. Consent forms included a specific clause allowing data from initial BBT participation to be linked to current data collection.

### Participants

Neuroimaging and clinical improvement data from 15 youth were analyzed for the current study. All BBT families initially recruited from the San Diego site (October 2010 to December 2014) who consented to being contacted in the future regarding additional opportunities for research were considered for participation in this neuroimaging follow-up (*N* = 49). Recruitment targeted participants randomized to the BBT arm to allow for inferences to be made regarding the association between treatment response to BBT and reward processing circuitry, as well as to control for treatment type and dose received. BBT caregivers of record were contacted via phone between August 2016 and November 2017 to assess interest as well as youth contraindications for undergoing an fMRI scan.

Of the 49 participants randomized to receive BBT through the San Diego site, 44 were contacted to assess interest in the current investigation (i.e., consented to further contact, not lost to follow-up by the RCT final assessment). Of those 44, four were lost to follow-up (e.g., contact information was out of date) and one participant had died since RCT study completion. We connected by phone with the remaining 39 participants to assess eligibility; 10 declined to participate, while 29 completed the phone screen. Of those screened, 21 met eligibility requirements for the neuroimaging follow-up (e.g., expressed interest in completing study activities, denied contraindications for the fMRI environment). Post-screen, three eligible participants were lost to follow-up and one declined to participate, prior to consenting to the current study. Thus, the current study enrolled 17 youth. Of the 17 consented to participate in the neuroimaging follow-up, one individual refused to complete the fMRI scan and one individual yielded an unusable dataset due to technical error. Table 1 reports sample characteristics of the 15 youth included in the current study’s analyses.

**TABLE 1:** Sample demographics.

***N***	**15**
**Age**	
Baseline	11.42 (1.64)
Time of scan	15.29 (2.42)
**Gender (% Female)**	7 (47%)
**Race**	
White	10 (67%)
Multiracial	4 (27%)
Other	1 (7%)
**Ethnicity (% Hispanic)**	5 (33%)
**Days between post-treatment and scan**	
Face Task	1284.20 (565.48)
Piñata Task	1325.93 (562.94)
**Face Task accuracy**	93.58% (6.35%)
**Face Task bias**	
Happy (ranged from −44.52 to 55.81)	5.80 (26.73)
Sad (ranged from −29.80 to 46.28)	2.80 (21.47)
Threatening (ranged from −57.37 to 27.26)	−7.11 (20.43)
**CGI-I**	
Clinical improvement	2.53 (1.13)
Treatment response (% Responders)	6 (40%)
**SCARED**	15.73 (10.24)
**MFQ**	11.99 (13.48)

*Continuous variables are displayed as *M* (*SD*); categorical variables are displayed as *N* (%). CGI-I: Clinical Global Impressions, Improvement Scale assigned post-treatment (Treatment Response: CGI-I ≤2); SCARED, Screen for Child Anxiety and Related Disorders completed at time of scan; MFQ, Mood and Feelings Questionnaire completed at time of scan.*

Participants enrolled in the neuroimaging follow-up were initially randomized at a mean age of 11.43 years (*SD* = 1.61; range: 8−14 years); at the time of scan, participants had a mean age of 15.29 years (*SD* = 2.42; range: 9−19 years). Primary diagnostic complaints at initial RCT enrollment were predominantly within the anxiety spectrum [*n* = 15; 35% (*n* = 6) Generalized Anxiety Disorder, 35% (*n* = 6) Separation Anxiety Disorder, 18% (*n* = 3) Social Phobia]; 29% (*n* = 5) had clinically elevated depression in addition to anxiety. In terms of treatment response, 41% (*n* = 7) of the participants engaged in the neuroimaging follow-up were characterized as BBT treatment responders at post-treatment.

Those enrolled in the neuroimaging follow-up did not significantly differ from those recruited in San Diego who were randomized to BBT but ineligible for the follow-up on any demographic or clinical indicators at baseline or post-treatment, with the exception of baseline clinical severity of internalizing symptoms. Those who did not engage in the neuroimaging follow-up had higher average severity scores at baseline (*M* = 4.44, *SD* = 0.88) compared to those who participated [*M* = 4.00, *SD* = 0.61; *t*(43.34) = 2.04, *p* = 0.048]. However, this difference, though statistically significant, does not reflect practical differences in clinical presentation. It is also notable that a smaller proportion of the neuroimaging sample was categorized as treatment responders at post (41%), versus rates of response observed in the BBT sample as a whole [57%; χ^2^(1) = 3.41, *p* = 0.065]. This difference was not statistically significant.

### Measures

#### Demographics

Caregivers provided updated demographic information at the time of scan (e.g., age, gender; see Table 1).

#### Clinical Characteristics

Clinical improvement was measured by the Clinical Global Impressions, Improvement Scale (CGI-I; Guy, 1976). The CGI-I is a 7-point, single item indicator of clinical change, such that lower scores represent improvement (1 = *very much improved*), while higher scores reflect clinical deterioration (7 = *very much worse*). Trained and reliable independent evaluators unaware of the participant’s treatment condition assigned a CGI-I score at the post-treatment assessment, capturing change in symptoms of anxiety and/or depression since the baseline assessment. The original trial utilized the CGI-I as an indicator of treatment response; participants with a score of 1 (very much improved) or 2 (much improved) were categorized as treatment responders, while those with a score of ≥3 (minimally improved) were categorized as non-responders (Weersing et al., 2017). The current study included the CGI-I dimensionally, as the main predictor of interest, to evaluate the association between clinical improvement and brain function.

The Schedule for Affective Disorders and Schizophrenia for School-Aged Children-Present and Lifetime Version (Kaufman et al., 1997) was used to determine eligibility at baseline (i.e., the presence of a primary anxiety or depression diagnosis). At the time of scan, caregivers completed the Screen for Child Anxiety and Related Disorders (SCARED; Birmaher et al., 1997) and the Mood and Feelings Questionnaire (MFQ; Angold et al., 1987) to inform severity of current anxiety and depression symptoms in youth, respectively, with higher scores indicating increased symptom severity (see Table 1). This study utilized the SCARED and MFQ in Additional Analyses intended to identify the impact of current symptoms on the observed pattern of fMRI findings.

#### Neuroimaging Paradigms

Youth completed two tasks that elicited neural activation within the context of social or non-social reward. Scans occurred 568−2317 days after the post-treatment assessment (see Table 1 and “Additional Analyses”). Participants completed the social reward task prior to the non-social reward task. This determination was made due to the social reward task lasting longer with greater potential for cognitive fatigue than the non-social reward task. As two participants provided data collected in the inverted order, task order was included as a potential confound (see section “Additional Analyses”).

##### Social reward task (Face Task)

Participants performed a jittered, event-related task with emotional face stimuli, including happy (i.e., social reward) faces, using a dot probe paradigm adapted from the Tel Aviv University/National Institute of Mental Health paradigm (Abend et al., 2014) during fMRI data acquisition. This task was modified to include four valence categories: happy, angry, sad, and neutral faces (see Supplementary Figure S1). Emotional faces were from the NimStim Set of Facial Expressions^1^ (Tottenham et al., 2009). Each trial began with a fixation cross for 500 ms. Next, neutral-neutral or neutral-emotional face pairs were presented on the screen for 500 ms, followed by a probe (< or >) presented for 1000 ms. The probe was positioned either in place of the emotional face (congruent condition) or the neutral face (incongruent condition). Participants were instructed to respond quickly and accurately by pressing the button that corresponded to the direction in which the probe was pointing (left or right). Inter-trial intervals were jittered (250−1180 ms, *M* = 715 ms).

The faces dot-probe paradigm was advantageous to control for potential differences in attention and to present social reward faces, in addition to faces reflecting other valences (angry, sad, neutral). These benefits allowed for the examination of specificity of response to the social reward faces. The inclusion of faces in dot-probe paradigms has been shown to probe areas implicated in reward processing in prior research in anxious (Shechner et al., 2012) and depressed (Forbes, 2011) youth.

Participants completed three runs of the Face Task (7 min. 27 s. per run). There were eight total conditions included in the task: happy-neutral/congruent, happy-neutral/incongruent, angry -neutral/congruent, angry-neutral/incongruent, sad-neutral/congruent, sad-neutral/incongruent, neutral-neutral/congruent, and neutral-neutral/incongruent. Neutral-neutral pairs were randomly split into “congruent” and “incongruent” groups for analysis purposes. Due to lack of jitter between face and probe displays, presentation could not be separated for analysis. There were 48 trials per condition. All participants had >65% accuracy and were therefore included in analyses (see Table 1). Of the 15 participants included in the Face Task analyses, 14 participants had three usable runs and one had two usable runs (excessive motion in the third run; see fMRI Data Processing).

##### Non-social reward task (Piñata Task)

A child-friendly monetary incentive delay task was utilized to assess neural functioning within the context of non-social reward (i.e., Piñata Task; see Supplementary Figure S2; Helfinstein et al., 2013; Wiggins et al., 2017; Dougherty et al., 2018). The Piñata Task is an event-related task (Helfinstein et al., 2013) previously used to reliably elicit reward-related brain activation in children (Wiggins et al., 2017). Each trial began with a variable length anticipation period consisting of a 2000 ms indicator of whether or not the participant had the opportunity to receive a reward during that round, followed by a 2500−5500 ms jittered delay period (together, referred to as the Cue Period). Then, the participant was presented with a target (i.e., turtle-shaped piñata) that they were instructed to “hit” (i.e., push a button to simulate striking the piñata). The fMRI operator explicitly instructed participants to attempt to hit the piñata during each trial, independent of reward condition. On reward trials, participants earned stars that translated into money earned (≤ $15), if they struck the piñata within the time limit. The time to hit the piñata was automatically adjusted in real time (+/−50 ms), based on the participant’s performance, to promote approximately 2/3 hit trials and 1/3 miss trials. If the participant pressed the button within the time allotted, the piñata broke, indicating a hit. Missed targets swung away (1500 ms). A basket was then shown displaying stars (reward/hit condition) or empty (reward/miss or no reward conditions; referred to as the Feedback Period, 1500 ms). Inter-trial intervals were jittered.

Participants completed three runs with a total of 60 trials across all runs (30 reward, 30 no reward conditions). Task runs spanned 4 min and 52 s. Non-social analyses included all available data from 14 participants, all of whom completed three runs of the task. The fifteenth participant was excluded from analyses due to data acquisition error.

## Analytic Plan

Three sets of analyses were completed: (a) evaluation of the association between clinical improvement and neural reactivity within the context of social reward (i.e., in the Face Task); (b) evaluation of improvement and neural reactivity within the context of non-social reward (i.e., monetary; Piñata Task); (c) direct comparison of social and non-social reward findings. Each set included group-level models that evaluated whole-brain activation, as well as seed-based functional connectivity. Although full factorial models were planned and executed, we focused on contrasts that included clinical improvement, given our interest in understanding that association between clinical improvement and neural processes. As such, direct comparisons of tasks were conducted if results by task yielded findings dependent on level of clinical improvement.

### Neuroimaging Acquisition

Functional and anatomical brain images were acquired using a 3T General Electric MRI scanner with a 32-channel head coil, with multiband procedures to increase spatial and temporal resolution and thus better infer correlates of clinical improvement. Task stimuli were projected onto a screen at the foot of the fMRI bed and seen by the participant via a mirror attached to the head coil. Participants responded to displayed stimuli using their dominant hand to manipulate a 2-button response box. T2 blood oxygen level dependent (BOLD) images were acquired across 3 runs as 104 interleaved sagittal slices approximately parallel to the AC-PC line, with whole-brain coverage using a 3D multiband EPI pulse sequence [matrix size = 104 × 104 × 60 accelerated by a factor of 6, TR = 800 ms, TE = 29 ms, flip angle = 52°, FOV = 20.8 mm, voxel size = 2 mm × 2 mm × 2 mm, 556 (Face Task) or 370 (Piñata Task) image volumes per run]. High-resolution anatomical images with prospective motion correction (T2-weighted MPRAGE PROMO) were acquired for anatomical localization and spatial normalization (256 1.0 mm sagittal slices, flip angle = 8°, matrix size = 256 × 256, FOV = 25.6 mm, voxel size = 1 mm × 1 mm × 1 mm). The acquisition protocol was not optimized to capture cerebellar signal, so clusters identified within the cerebellum will not be discussed in detail.

### fMRI Data Preprocessing

Preprocessing protocols were implemented using Analysis of Functional NeuroImages (AFNI^2^). Preprocessing steps included functional image realignment, slice-time correction, spatial smoothing of 4 mm, and non-linear registration for spatial standardization to the Talairach template (Talairach and Tournoux, 1988). Image volume pairs with frame-wise displacement >1 mm were censored from individual level analysis. Task runs with censoring of ≥35% of image volumes were excluded from analyses (Face Task: 1 run of 1 participant). All participants evidenced mean frame-wise displacement (head motion) ≤0.30 mm.

### Data Analysis

#### Activation

For the Face Task, probing social reward, regressors of interest in the individual-level general linear model included face emotion (happy, sad, angry, neutral) and probe location (congruent, incongruent), convolved with the BLOCK function. Beta images represented estimated activation during each of the conditions.

For the Piñata Task, probing non-social reward, two individual-level general linear models were run to generate estimates of brain activation during anticipation and feedback periods, separately. The regressor of interest during the anticipation period included Reward Condition (no reward, reward). Reward Condition was convolved with AFNI’s “dmBLOCK” basis function over the variable duration. Regressors of interest during the feedback period included Reward Condition and Performance (hit, miss). Both were convolved with the “BLOCK” function over 1500 ms. Analyses generated beta coefficients at each voxel for reward and no reward trials during the anticipation period, as well as for reward/hit, reward/miss, no reward/hit, and no reward/miss trials during the feedback period.

For both the social reward and non-social reward tasks, models included head motion in x, y, z, roll, pitch, yaw directions and third-degree polynomials to model low-frequency drift as nuisance regressors.

#### Connectivity

Generalized psychophysiological interaction analysis (gPPI; McLaren et al., 2012) was utilized to calculate functional connectivity during the Face Task and the feedback period of the Piñata Task, given prior work that found connectivity results in the feedback period (Dougherty et al., 2018). gPPI calculates change in correlations between a seed region of interest and all other brain regions in each condition compared to implicit baseline. gPPI is advantageous as it allows for the evaluation of more than two task conditions in a single model. Given past work on reward tasks (Helfinstein et al., 2013; Dougherty et al., 2014; Wiggins et al., 2017) and prior fMRI work on anxiety (e.g., Blackford and Pine, 2012) and depression (e.g., Kerestes et al., 2014), bilateral amygdalae and ventral striatum (nucleus accumbens) were utilized as seeds for gPPI analyses. Seed regions were identified using the Talairach atlas in AFNI (Talairach and Tournoux, 1988; left amygdala = 1288 mm^3^; right amygdala = 1280 mm^3^; left ventral striatum = 136 mm^3^; right ventral striatum = 168 mm^3^). This analysis generated a set of voxel-wise images that represent connectivity between the seed region and the rest of the brain per condition for each task. Variance associated with head motion in x, y, z, roll, pitch, yaw directions was removed and third-degree polynomials were included to remove low-frequency drift.

#### Second Level Analyses

We conducted whole-brain, group-level ANCOVAs, by task, via AFNI’s 3dMVM program to evaluate the association between clinical improvement (CGI-I) and reward-related brain function (activation, connectivity). Clinical improvement was included as a dimensional, between-subjects variable; task conditions were included as within-subjects categorical variables. Interactions between clinical improvement and task condition (Face Task: Clinical Improvement × Face Emotion, Clinical Improvement × Face Emotion × Probe Location; Piñata Task: Clinical Improvement × Reward Condition, Clinical Improvement × Performance, Clinical Improvement × Reward Condition × Performance) indicate the impact of task condition(s) on the relationship between clinical improvement and brain function.

Due to the small sample size, 3-way interactions were considered exploratory and interpreted with caution. All results were corrected for multiple comparisons, with a whole-brain corrected threshold of *p* < 0.05. The cluster threshold was calculated by 3dClustsim using the mixed-model spatial autocorrelation function (-acf) and the NN1 2-sided option, per the most recent recommendations on cluster correction (Cox et al., 2017). 3dClustsim used a group mask representing brain regions where 90% of participants had valid data. Spatial autocorrelation parameters were calculated by 3dFWHMx for each run separately, averaged over runs for each participant, and then averaged across participants. The cluster extent threshold across all models was *k* ≥ 56 voxels with a conservative height threshold of *p* < 0.005, which is appropriate for event-related designs (Cox et al., 2017). To decompose significant interactions, *post hoc* analyses were performed on values that were extracted and averaged from each cluster using SPSS; *z*-scores represented the test of the difference between two dependent correlations with one variable in common (Steiger, 1980; Lee and Preacher, 2013). *Post hoc* correlations were conducted for illustrative purposes to clearly depict the direction of effects.

#### Additional Analyses

Activation/connectivity values were extracted from clusters representing the main results and averaged for Additional Analyses. Regression analyses evaluated the effects of residual head motion, age, gender, concurrent anxiety, concurrent depression, length of time since post-treatment assessment, and task order on identified findings.

## Results

### Behavioral Results

On the Face Task, overall mean accuracy (*M* = 93.58%, *SD* = 6.35) was well above chance (50%). Attention bias was calculated by subtracting reaction time between congruent and incongruent trials within face emotion condition. One-sample *t*-tests revealed no significant attention bias toward happy [*t*(14) = 0.84, *p* = 0.415], sad [*t*(14) = 0.51, *p* = 0.621], or threatening [*t*(14) = −1.35, *p* = 0.199] faces. No significant associations were found between clinical improvement and task accuracy (*r* = 0.29, *p* = 0.299) or bias scores (*r*_happy_ = −0.21, *p* = 0.453; *r*_sad_ = 0.26, *p* = 0.354; *r*_threatening_ = −0.31, *p* = 0.257).

### fMRI Social Reward/Face Task

#### Activation

**FIGURE 1:**
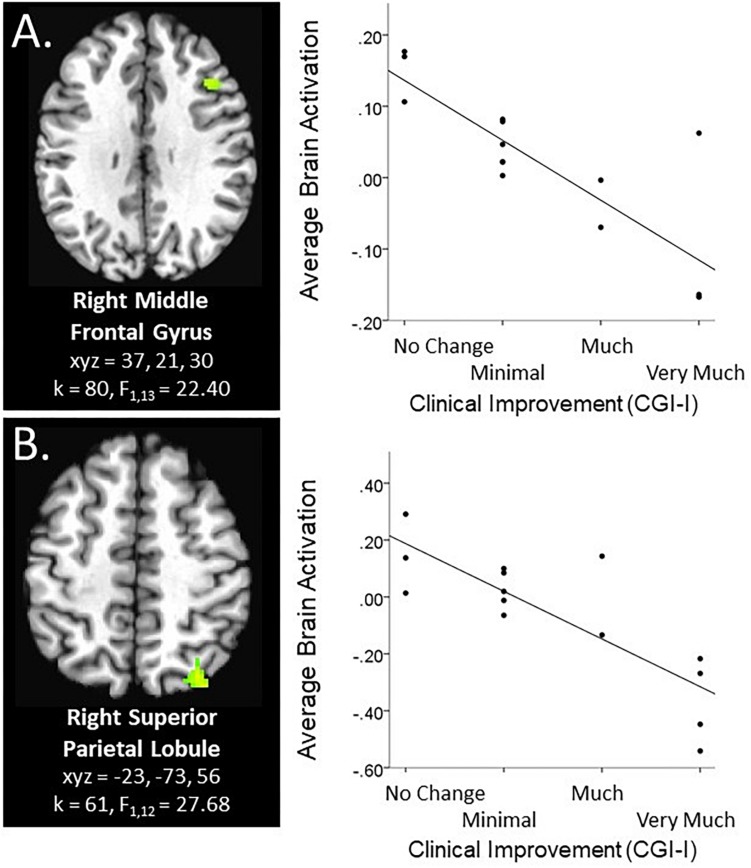
Main effect of clinical improvement during Social and Non-social reward tasks. Scatterplots of significant brain activation averaged across all task conditions in the specified paradigm represent the main effect of CGI-I in **(A)** the right middle frontal gyrus during the social reward paradigm (Face Task; *N* = 15), and **(B)** the right superior parietal lobule during the anticipation phase of the non-social reward paradigm (Piñata Task; *N* = 14). Brain images represent axial sections (left = left) with threshold set at whole-brain FDR-corrected *p* < 0.05. Scatterplots are displayed for illustrative purposes to depict the direction of association. Additionally, CGI-I (i.e., clinical improvement; Guy, 1976) anchors are displayed inversely to improve readability and restricted to reflect the range observed within this dataset.

Within the context of the Face Task, there was a significant main effect of clinical improvement in the right middle frontal gyrus, such that across emotional faces (including social reward faces), decreased activation in this region related to a greater degree of clinical improvement (see Figure 1A). Additional significant activation clusters were evidenced in the cerebellum, as a function of degree of clinical improvement (see Table 2).

**TABLE 2:** Significant clusters resulting from whole-brain analyses evaluating the association between clinical improvement and neural reactivity within the context of social reward (*N* = 15).

***k***	***F***	***x***	***y***	***z***	**BA**	**Region**
**Whole-brain activation**						
*^∗^CGI-I main effect (df = 1, 13)*						
80^+^	22.4	37	21	30	9	Right middle frontal gyrus
**Whole-brain left ventral striatum connectivity**						
*CGI-I × probe location (df = 1, 13)*						
70	29.6	61	−37	−2	21	Right middle temporal gyrus
*^∗^CGI-I main effect (df = 1, 13)*						
90^+^	30.4	−7	−53	4	19	Left lingual gyrus
77^+^	30.4	−1	−61	44	7	Left precuneus
76^+^	25.0	17	−71	6	18	Right lingual gyrus/ right cuneus
75^+^	27.4	−7	−91	−4	17	Left lingual gyrus
58^+^	47.3	−23	−7	−4	N/A	Left dorsal striatum
**Whole-brain right ventral striatum connectivity**						
*^∗^CGI-I main effect (df = 1, 13)*						
163	36.0	−17	−29	60	2, 3, 4	Left postcentral gyrus/ left precentral gyrus
81^+^	24.2	−29	−5	56	6	Left middle frontal gyrus/ left precentral gyrus
75^+^	45.4	−17	−61	52	7	Left precuneus
74	30.4	43	−31	56	40	Right postcentral gyrus
59	33.2	−29	−65	26	39	Left angular gyrus
59	37.8	33	−33	38	40	Right inferior parietal lobule
**Whole-brain left amygdala connectivity**						
*^∗^CGI-I × face emotion (df = 3, 39)*						
73	10.4	65	−17	26	2	Right postcentral gyrus
**Whole-brain right amygdala connectivity**						
*^∗^CGI-I × face emotion (df = 3, 39)*						
98	12.8	11	−73	−20	N/A	Right declive/ right cerebellum
71	9.0	−13	−75	−40	N/A	Left inferior semi-lunar lobule/ left cerebellum
*CGI-I × probe location (df = 1, 13)*						
148	42.2	47	33	26	9	Right middle frontal gyrus
*Probe location main effect (df = 1, 13)*						
149	36.3	−23	45	24	10	Left superior frontal gyrus/ left middle frontal gyrus

**FIGURE 2:**
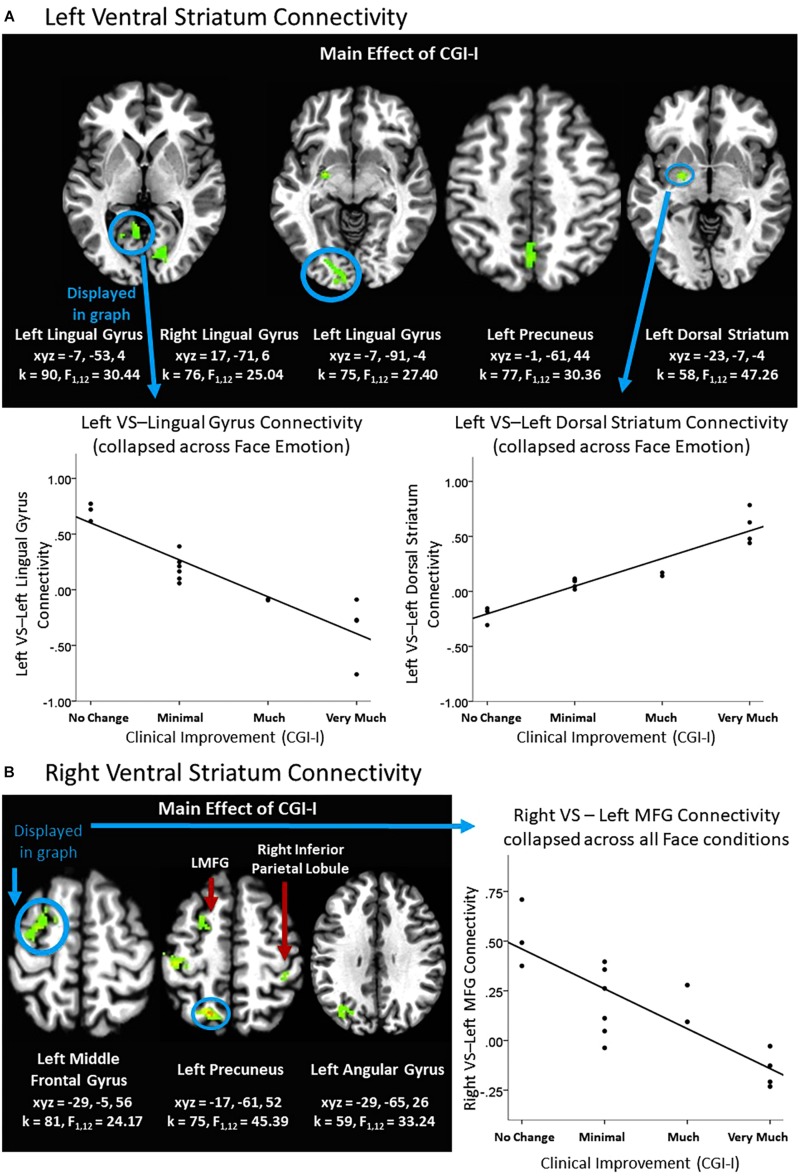
Effects of clinical improvement on ventral striatum connectivity within the context of social reward. **(A)** Left ventral striatum (VS) connectivity; **(B)** Right ventral striatum connectivity. *N* = 15. Brain images represent axial sections (left = left) with threshold set at whole-brain FDR-corrected *p* < 0.05. Scatterplots of the significant connectivity effects for the indicated clusters are displayed for illustrative purposes to depict the direction of association; patterns are similar for other clusters within each contrast. CGI-I (i.e., clinical improvement; Guy, 1976) anchors are displayed inversely to improve readability and restricted to reflect the range observed within this dataset. L, left; MFG, middle frontal gyrus; R, right; TPJ, temporo-parietal junction.

*Contrasts that did not yield significant clusters are not listed in this table. ^∗^ indicates a contrast of interest; ^+^ indicates clusters from which values were extracted and presented in Figures 1, 2. BA, brodmann area; CGI-I, Clinical Global Impressions, Improvement Scale (Guy, 1976).*

#### Connectivity

There was a main effect of clinical improvement on right and left ventral striatum connectivity with multiple posterior regions in the Face Task, including lingual gyrus, precuneus, angular gyrus, as well as middle frontal gyrus, inferior parietal lobule, and dorsal striatum (see Figure 2). Across all clusters, clinical improvement was associated with less connectivity between the ventral striatum and posterior regions, with the exception of the left ventral striatum to left dorsal striatum connectivity. Within that cluster, clinical improvement was associated with increased connectivity (see Figure 2A). Additionally, clusters representing right amygdala connectivity with cerebellum were significant for Clinical Improvement × Face Emotion (see Table 2). Furthermore, the Clinical Improvement × Face Emotion interaction yielded significant left amygdala and post-central gyrus connectivity (see Table 2); however, *post hoc* analyses revealed that this cluster does not survive *post hoc* evaluation for potential confounding variables and may be outlier-driven.

##### Exploratory connectivity analysis

**FIGURE 3:**
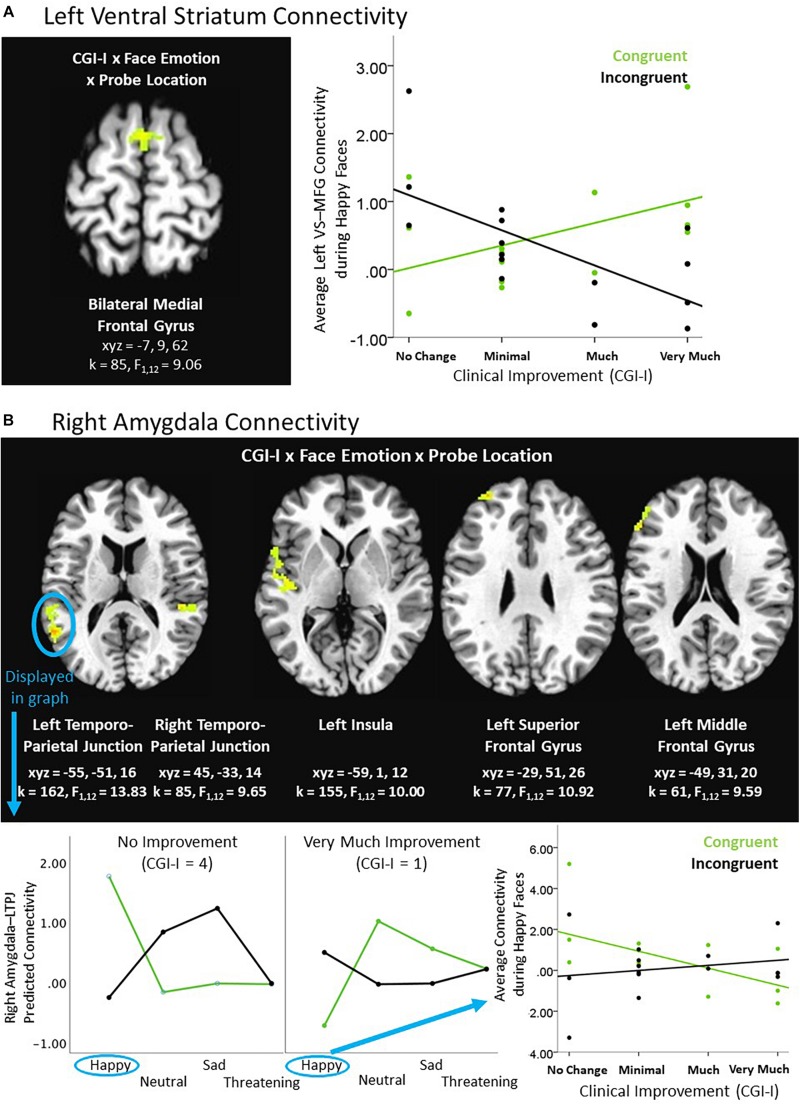
Exploratory higher level interactive effects of clinical improvement on ventral striatum and amygdala connectivity within the context of social reward. **(A)** Left ventral striatum (VS) connectivity; **(B)** Right amygdala connectivity. *N* = 15. Brain images represent axial sections (left = left) with threshold set at whole-brain FDR-corrected *p* < 0.05. Scatterplots of the significant connectivity effects for the indicated clusters are displayed for illustrative purposes to depict the direction of association; patterns are similar for other clusters within each contrast. CGI-I (i.e., clinical improvement; Guy, 1976) anchors are displayed inversely to improve readability and restricted to reflect the range observed within this dataset. L, left; MFG, middle frontal gyrus; R, right; TPJ, temporo-parietal junction.

An exploratory Clinical Improvement × Face Emotion × Probe Location interaction in the social reward task revealed significant connectivity between the left ventral striatum and medial frontal gyrus. *Post hoc* analyses indicated that the ventral striatum-medial frontal connectivity cluster was driven by differences in response to happy faces (i.e., the social reward stimulus). Heightened clinical improvement was associated with greater connectivity during happy/congruent trials; however, less connectivity was observed during happy/incongruent trials (see Table 3 and Figure 3A). Similarly, using the right amygdala as the seed-region of interest, the 3-way interaction evidenced significant connectivity between the right amygdala and multiple temporal and frontal clusters, including bilateral temporo-parietal junction, insula, and prefrontal cortex. Specifically, the magnitude of right amygdala connectivity with these temporal and frontal regions differed as a function of degree of clinical improvement and depended on face emotion type, as well as congruency. Like findings in the ventral striatum, these interactions were driven by social reward. Participants evidencing less clinical improvement in response to BBT exhibited greater connectivity during happy/congruent trials compared to happy/incongruent trials. In contrast, participants with the most clinical improvement evidenced the opposite pattern: greater connectivity during happy/incongruent trials compared to happy/congruent trials. *Post hoc* analyses supported that correlations between clinical improvement and amygdala connectivity for happy/congruent vs. happy/incongruent trials differed significantly in all clusters (see Figure 3B).

**TABLE 3:** Significant clusters resulting from exploratory 3-way interactions evaluating the association between clinical improvement and neural reactivity within the context of social reward (*N* = 15).

***k***	***F***	***x***	***y***	***z***	**BA**	**Region**
**Whole-brain activation**						
*CGI-I × face emotion × probe location (df = 3, 39)*						
154	12.4	1	−67	−22	N/A	Right pyramis/ right cerebellum
77	7.9	−37	−69	−40	N/A	Inferior semi-lunar lobule
**Whole-brain left ventral striatum connectivity**						
*CGI-I × face emotion × probe location (df = 3, 39)*						
85^+^	9.1	−7	9	62	6	Bilateral medial frontal gyrus/ left superior frontal gyrus
71^∗∗^	10.2	−13	45	42	8	Left medial frontal gyrus/ left superior frontal gyrus
**Whole-brain right ventral striatum connectivity**						
*CGI-I × face emotion × probe location (df = 3, 39)*						
81	14.1	−1	−61	−18	N/A	Bilateral declive/ bilateral culmen/ cerebellar vermis/ left cerebellum
73	9.0	3	−39	−38	N/A	Left cerebellar tonsil/ left cerebellum/ lobule IX
**Whole-brain right amygdala connectivity**						
*CGI-I × face emotion × probe location (df = 3, 39)*						
162^+^	13.8	−55	−51	16	39, 22, 13	Left temporo-parietal junction
155^+^	10.0	−59	1	12	22, 13, 6	Left insula/ left superior temporal gyrus/ left precentral gyrus
85^+^	9.6	45	−33	14	41	Right temporo-parietal junction
77^+^	10.9	−29	51	26	9	Left superior frontal gyrus
61^+^	9.6	−49	31	20	46	Left middle frontal gyrus
58	10.1	37	−71	−32	N/A	Right pyramis/ right cerebellum

*Contrasts that did not yield significant clusters are not listed in this table. ^∗∗^ indicates findings not primarily driven by brain responses to happy faces; ^+^ indicates clusters from which values were extracted and presented in Figure 3. BA, brodmann area; CGI-I, Clinical Global Impressions, Improvement Scale (Guy, 1976).*

Of note, there were additional significant clusters for the Clinical Improvement × Probe Location (see Table 2) and the Clinical Improvement × Face Emotion × Probe Location (see Table 3) contrasts, but these interactions were not driven by social reward faces.

### fMRI Non-social Reward/Piñata Task

#### Activation

Within the context of the Piñata Task, during the anticipation period, increased clinical improvement related to decreased activation in the superior parietal lobule across reward and no reward conditions (see Figure 1B). During the feedback period of the Piñata Task, activation significantly varied as a function of task conditions; however, contrasts that included clinical improvement were not statistically significant. Additional significant activation clusters are reported in Table 4.

**TABLE 4:** Significant clusters resulting from whole-brain analyses evaluating the association between clinical improvement and neural reactivity within the context of non-social reward (*N* = 14).

***k***	***F***	***x***	***y***	***z***	**BA**	**Region**
**Whole-brain activation: cue period**						
*^∗^CGI-I main effect (df = 1, 12)*						
62	24.3	23	−67	−42	N/A	Right inferior semilunar lobule
61^+^	27.7	23	−73	56	7	Right superior parietal lobule
*Reward condition main effect (df = 1, 12)*						
301	100	31	−77	18	19	Right middle occipital gyrus
240	40.6	−29	−83	18	19	Middle occipital gyrus
153	36.9	−27	−65	−4	19	Left declive
146	34.7	23	−67	32	7	Right precuneus
88	37.4	−17	−71	38	7	Left precuneus
82	38.5	55	−27	6	22	Right superior temporal gyrus
**Whole-brain activation: feedback period**						
*Reward condition main effect (df = 1, 12)*						
79	40.9	1	33	34	6	Right medial frontal gyrus
*Performance main effect (df = 1, 12)*						
2047	100.0	9	−81	6	18	Lingual gyrus
1657	100.0	−1	31	24	32	Right medial frontal gyrus
841	96.0	53	−45	12	22	Right superior temporal gyrus
733	100.0	37	17	8	13	Right insula
370	67.2	35	5	34	6	Right precentral gyrus
275	99.0	−31	11	14	13	Left insula
262	40.5	−5	−27	30	23	Left cingulate gyrus
253	64.3	33	39	30	10	Right middle frontal gyrus
173	32.1	−25	51	22	10	Left superior frontal gyrus
160	56.5	37	−37	−6	19	Right parahippocampal gyrus
136	53.4	3	−47	44	7	Right precuneus
122	24.9	39	59	12	10	Right middle frontal gyrus
101	44.5	9	−11	8	N/A	Right thalamus
89	34.2	−33	−51	−30	N/A	Left culmen
62	29.0	−19	−33	32	N/A	Left cingulate gyrus
**Whole-brain left ventral striatum connectivity: feedback period**						
*^∗^CGI-I × reward condition (df = 1, 12)*						
69^+^	41.5	23	47	2	10	Right superior frontal gyrus
68^+^	32.4	9	33	−2	32	Right anterior cingulate
**Whole-brain right ventral striatum connectivity: feedback period**						
*^∗^CGI-I × reward condition (df = 1, 12)*						
99^+^	31.1	41	−63	32	7, 39	Right superior parietal lobule/ right inferior parietal lobule
62^+^	34.6	−33	−71	42	19, 7	Left precuneus/ left superior parietal lobule
*Reward condition x performance (df = 1, 12)*						
406	42.1	37	47	22	10	Right middle frontal gyrus/ right superior frontal gyrus
129	32.1	31	11	52	6, 8	Right superior frontal gyrus/ right middle frontal gyrus
*^∗^CGI-I main effect (df = 1, 12)*						
65^+^	23.6	47	51	10	10	Right middle frontal gyrus
**Whole-brain left amygdala connectivity: feedback period**						
*^∗^CGI-I × reward condition (df = 1, 12)*						
87	52.3	33	−93	4	18	Right middle occipital gyrus
**Whole-brain right amygdala connectivity: feedback period**						
*^∗^CGI-I × reward condition (df = 1, 12)*						
80	45.3	−39	−1	26	6	Left precentral gyrus
*Reward condition main effect (df = 1, 12)*						
57	50.2	7	−53	32	31	Right precuneus

**FIGURE 4:**
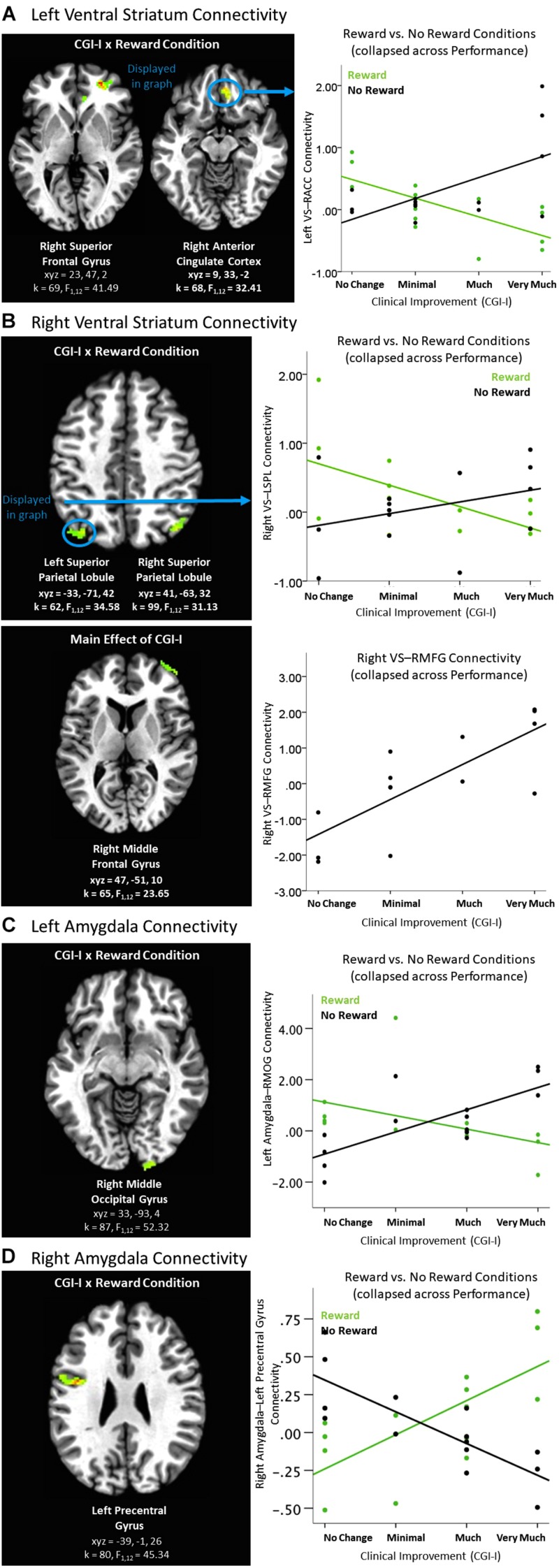
Effects of clinical improvement on ventral striatum and amygdala connectivity when receiving feedback within the context of non-social reward. **(A)** Left ventral striatum (VS) connectivity; **(B)** Right ventral striatum connectivity; **(C)** Left amygdala connectivity; **(D)** Right amygdala connectivity. *N* = 14. Brain images represent axial sections (left = left) with threshold set at whole-brain FDR-corrected *p* < 0.05. Scatterplots of the significant connectivity effects for the indicated clusters are displayed for illustrative purposes to depict the direction of association; patterns are similar for other clusters within each contrast. CGI-I (i.e., clinical improvement; Guy, 1976) anchors are displayed inversely to improve readability and restricted to reflect the range observed within this dataset. ACC, anterior cingulate cortex; L, left; MFG, middle frontal gyrus; MOG, middle occipital gyrus, R, right; SPL, superior parietal lobule; TPJ, temporo-parietal junction.

*Contrasts that did not yield significant clusters are not listed in this table. ^∗^ indicates a contrast of interest; ^+^ indicates clusters from which values were extracted and presented in Figures 1, 4. BA, brodmann area; CGI-I, Clinical Global Impressions, Improvement Scale (Guy, 1976).*

#### Connectivity

Differences in connectivity as a function of degree of clinical improvement were evident during the Feedback period of the Piñata task. When the ventral striatum was used as the seed region of interest, left and right ventral striatum connectivity analyses yielded significant clusters in multiple medial prefrontal and parietal regions for the Clinical Improvement × Reward Condition interaction during the Feedback period (see Table 4 and Figures 4A,B). Across all of these clusters, greater clinical improvement was associated with less connectivity during reward conditions yet greater connectivity during the no reward conditions. In addition, greater connectivity between the right ventral striatum and right middle frontal gyrus was observed across reward conditions (see Figure 4B). The Clinical Improvement x Reward Condition interaction also yielded significant clusters reflecting connectivity between the left amygdala and right middle occipital gyrus and between the right amygdala and the left precentral gyrus (see Figures 4C,D). *Post hoc* analyses within these clusters supported that greater clinical improvement was associated with less connectivity between the left amygdala and right middle occipital gyrus during reward conditions yet greater connectivity between these regions during the no reward conditions across clusters. However, the connectivity patterns between the right amygdala and left precentral gyrus evidenced the opposite pattern of findings.

##### Exploratory connectivity analysis

**FIGURE 5:**
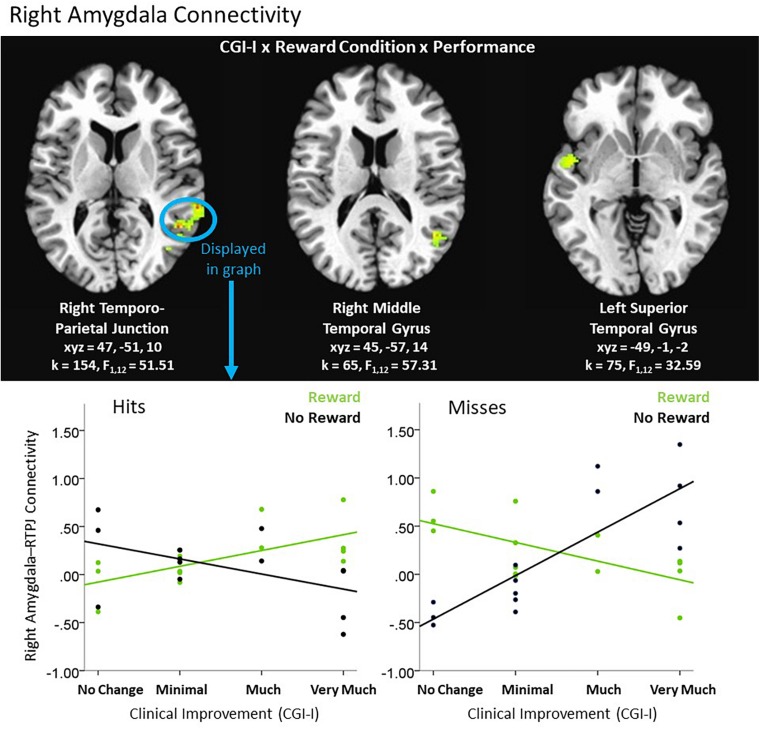
Exploratory higher level interactive effects of clinical improvement on right amygdala connectivity when receiving feedback within the context of non-social reward. *N* = 14. Brain images represent axial sections (left = left) with threshold set at whole-brain FDR-corrected *p* < 0.05. Scatterplots of the significant connectivity effects for the indicated clusters are displayed for illustrative purposes to depict the direction of association; patterns are similar for other clusters within each contrast. CGI-I (i.e., clinical improvement; Guy, 1976) anchors are displayed inversely to improve readability and restricted to reflect the range observed within this dataset. R, right; TPJ, temporo-parietal junction.

An exploratory Clinical Improvement x Reward Condition x Performance interaction during the feedback period of the Piñata Task revealed significant left and right amygdala connectivity with multiple temporal and parietal regions, including temporal-parietal junction (see Table 5). In these regions, increased clinical improvement was associated with greater amygdala connectivity when participants either received a reward for hitting the target (i.e., reward/hit condition) or missed the target when no reward was expected (i.e., no reward/miss condition). In contrast, increased clinical improvement was associated with lower levels of amygdala connectivity when participants either hit the target but did not receive a reward (i.e., no reward/hit condition) or missed the target when a reward was expected (i.e., reward/miss condition). *Post hoc* analyses indicated that the relationship between clinical improvement and brain activation in reward vs. no reward conditions differed significantly for both hit and miss trials, across all clusters (see Figure 5).

**TABLE 5:** Significant clusters resulting from exploratory 3-way interactions evaluating the association between clinical improvement and neural reactivity within the context of non-social reward (*N* = 14).

***k***	***F***	***x***	***y***	***z***	**BA**	**Region**
**Whole-brain left amygdala connectivity: feedback period**						
*CGI-I × reward condition × performance (df = 1, 12)*						
152	63.0	−29	−57	46	7	Superior parietal lobule^a^
**Whole-brain right amygdala connectivity: feedback period**						
*CGI-I × reward condition × performance (df = 1, 12)*						
154^+^	51.5	47	−51	10	22	Right temporo-parietal junction
75^+^	32.6	−49	−1	−2	22	Left superior temporal gyrus
65^+^	57.3	45	−57	14	39, 22	Right middle temporal gyrus
59	47.5	5	−23	−2	N/A	Right brainstem/ right thalamus

*Contrasts that did not yield significant clusters are not listed in this table. ^*a*^ indicates a cluster that failed to maintain significance when controlling for parent-rated youth anxiety, irritability, or depression. ^+^ indicates clusters from which values were extracted and presented in Figure 5. BA, brodmann area; CGI-I, Clinical Global Impressions, Improvement Scale (Guy, 1976).*

### Comparison of Findings by Task Type

Our findings implicated connectivity differences during happy-neutral/congruent vs. happy-neutral/incongruent conditions in the Face Task and during reward/hit vs. reward/miss conditions in the Piñata Task. As such, we conducted an exploratory whole-brain ANCOVA to directly compare connectivity within the context of social vs. non-social reward, in relation to clinical improvement as a function of treatment. Contrast images, representing happy/congruent vs. happy/incongruent and reward/hit vs. reward/miss connectivity for left and right amygdalae as well as left and right ventral striata, were calculated and entered into separate 3dMVM models. Task type (social reward, non-social reward) was included as a categorical, within-subjects variable and clinical improvement was maintained as a dimensional, between-subjects variable.

#### Activation

We were unable to statistically evaluate differences in whole-brain activation across tasks due to a lack of findings dependent on clinical improvement scores during the feedback period of the Piñata Task.

#### Connectivity

Clinical Improvement × Task Type (i.e., social reward, non-social reward) did not yield significant clusters indicative of connectivity between bilateral amygdalae or ventral striata and other regions in the brain.

### Additional Analyses

Additional analyses were run to evaluate the impact of potential confounding factors on the reported results. Hypothesized confounds utilized in these analyses included residual head motion, age, gender, concurrent anxiety, concurrent depression, length of time since post-treatment assessment, and task order. All main results maintained significance after the inclusion of these factors in statistical models.

## Discussion

The current study aimed to add to the sparse literature on reward processing in pediatric anxiety and depression. Of note, the focus of this work was to evaluate neural correlates of a successful unified protocol, rather than to determine exclusive and common correlates of depression and anxiety in youth. Future research can build on the results of our work to distinguish neural correlates in these highly comorbid conditions. This investigation is unique in its focus on social as well as non-social reward tasks, use of a clinically impaired service-seeking sample, and effort to probe associations between reward and clinical improvement in a treated sample. Indeed, this study is the only study to investigate reward processing in a treatment context within a sample of anxious youth and one of only four to do so in pediatric depression. Moreover, this was the only study to investigate multiple tasks (i.e., social and non-social) within the same individuals, enabling the evaluation of task context effects. The study design was exploratory and hypothesis-generating in nature; indeed, findings should be interpreted cautiously due to the small sample size. Perhaps the most important message from this work is that evaluating the relationship between clinical improvement and reward processing neural circuitry, particularly within the context of transdiagnostic samples and treatment paradigms, appears promising for further investigation. Our findings indicate that integrating behavioral neuroscience tools into clinical science can provide a complementary way to “look under the hood” of treatments, and thus inform the generation of more targeted, mechanistically based treatments. Broadly, we found that social and non-social reward paradigms triggered patterns of connectivity in frontal and striatal regions associated with social cognition and reward, and these patterns reliably differed by level of clinical improvement obtained over the course of treatment. Patterns were consistent with the literature at large and suggest value in further efforts to probe shifts in reward processing as a mechanism of treatment-related changes in internalizing pathology in youth.

Findings further illustrate how data from multiple within-subject tasks can be incorporated in a single study and underscore the importance of task context for interpreting differences in brain function. Although both reward and emotion regulation networks were implicated in both the social and non-social reward tasks, the specific regions as well as the direction of differences (e.g., greater vs. less activation/connectivity) depended on task context and conditions. Thus, when summarizing the literature, it may not be enough to state that observed differences in activation or connectivity of a particular region is associated with a disease state, or that reducing or increasing brain function in those regions is the mechanism by which clinical improvement occurs. Rather, our study which compares and contrasts results from two tasks within the same participants suggests that differences in activation or connectivity appear to be highly dependent on task context. More direct comparisons of neural responses to social and non-social feedback, particularly in larger samples, are needed in future work, as findings have implications for the future of clinical care and evaluation of treatment mechanisms.

Although the specific patterns of results differed by task, clinical improvement was associated with alterations in regions involved in emotion regulation, in addition to reward, across both tasks. Observed differences as a function of degree of clinical improvement could reflect “normalization” of circuitry or compensatory mechanisms. This finding has intriguing implications for understanding the integration of emotion regulation and reward processing in treatment. As stated above, both anxious and depressed samples of youth have evidenced aberrant patterns of reward processing and emotion regulation, compared to healthy peers. The guided behavioral activation in concert with targeted skill building included in BBT may have interacted more effectively with reward circuitry compared to the more varied care received by those in ARC. Thus, successful treatment of internalizing pathology may require integration of emotion regulation and reward processing regions, such that youth are able to learn to modulate their emotions – which may in itself be rewarding. Improved emotion regulation skills and exposure in this treatment may have been sufficient to alter both reward and emotion regulation circuitry without an overt cognitive component.

Of note, significant contrasts in the Face Task were driven by the social reward stimulus (i.e., happy faces) in relation to clinical improvement. This is particularly remarkable given that prior work documented that across the lifespan, faces as social reward have been shown to be less impactful in motivating behavior change than monetary incentives (Kohls et al., 2009). Furthermore, the employed tasks were not perfectly parallel in terms of their assessment of social vs. non-social reward. The Face Task paradigm involved the passive viewing of rewarding faces, in contrast to the Piñata Task’s dependence on participant action to trigger the reward. Nevertheless, stronger connectivity between the amygdala and multiple frontal and temporal areas implicated in reward-based learning, social prediction error, and reappraisal of emotions triggered by social situations (e.g., Grecucci et al., 2013); but, weaker coupling between the ventral striatum and medial frontal gyrus, another area implicated in social cognition (Amodio and Frith, 2006) was observed when the Face Task probe was located under the neutral rather than happy face (i.e., incongruent trial). BBT’s focus on exposure may improve anxiety/depression symptoms by “incentivizing” engagement in adaptive activities, including social interactions; that is, repeated exposure and associated habituation may help participants re-define activities deemed dangerous as inherently rewarding, through the practice of approach behaviors and emotion regulation skills (i.e., a behavioral activation model). In addition, increased clinical improvement was associated with decreased activation in areas associated with re-orienting attention (Japee et al., 2015) and emotion reappraisal (Grecucci et al., 2013). This may moreover suggest that those who improved as a function of BBT may be less prone to distraction by internal processes (e.g., rumination, worry) and more able to maintain focus on their environment, perhaps supporting increased approach behaviors. Whereas we have taken this initial step of comparing tasks, future research can add to the literature base describing these relations using more parallel social vs. non-social task paradigms.

Within pediatric anxiety (e.g., Guyer et al., 2008) and depression (e.g., Silk et al., 2012), social reward processing has been a research target due to the characteristic fears of social evaluation by peers in internalizing youth. Evidence has suggested that youth exhibit a negative bias during social interactions, interpreting peer behavior as overly critical and expecting interactions to be negative in support of their misappraisal. Our findings with social reward stimuli (i.e., happy faces) are consistent with the idea that clinical improvement may occur through amelioration of this negative bias and greater valuation of social reward. Nevertheless, faces are relatively passive stimuli to assess reward processing, and the faces utilized by this task reflect adults, rather than same-aged peers. Moving forward, studies that build on our findings to further investigate social reward processing as a mechanism of treatment response may wish to consider more interactive tasks (e.g., Chatroom Task, Guyer et al., 2008; Virtual School, Jarcho et al., 2013) that include age-appropriate faces and peer evaluation as feedback, as these may be more ecologically valid for adolescents.

Within the context of non-social reward, differential coupling of the affective and cognitive control networks by condition was apparent. Increased clinical improvement was associated with increased connectivity between the amygdala and areas associated with cognitive control and decision making after positive or neutral experiences (e.g., obtaining a reward, missing when no reward was promised). In contrast, the same regions evidenced decreased connectivity in response to frustrating experiences (e.g., hitting the target when no reward was promised, missing the target when a reward was available). This may suggest more effective recruitment of emotion regulation strategies (e.g., decreased influence of emotional responses in decision making after aversive events) so as to maintain adaptive behavior. Interestingly, the areas implicated in non-social reward connectivity analyses also have been shown to be part of social cognition circuitry. As similar regions emerged across tasks, this serves as support that both tasks probe reward-related processes, which may in turn underlie clinical improvement.

Several limitations warrant consideration. First, although our sample size (*N* = 15) is comparable to that of the few other pediatric studies probing associations between neural mechanisms of reward processing and treatment response (e.g., *n* = 10, Straub et al., 2015; *n* = 13, Forbes et al., 2010; *n* = 15, Mori et al., 2016), our sample size was modest and represents a fraction of the participants who originally participated in the clinical trial (similar to comparable studies as well). Thus, our power was limited and our findings may not generalize to the broader population of youth. Replicating findings within a larger sample could strengthen interpretations and power more complex statistical models. Furthermore, the lack of interest in re-engaging in research suggests potential for sampling bias, as there may be differences between those who completed their scans and those who refused on dimensions not measured by the current battery. Nevertheless, given the paucity of studies characterizing neural treatment mechanisms, and as the only study to include multiple tasks within youth, this study serves as a proof-of-concept for future work.

Second, scanning occurred post-treatment, which limits our ability to determine whether the neural profiles identified are present prior to or as a consequence of clinical improvement. It is possible that our findings reflect that clinical improvement promoted the observed patterns of brain reactivity, suggesting that brain patterns were in fact outcomes of response. However, it is also possible that the observed brain patterns were pre-existing and therefore predictors of treatment outcome. A third option is that clinical improvement and observed brain patterns were both related to a third, unmeasured variable. Additionally, substantial time passed between treatment completion and the scan. Although the additional analyses suggested that our findings primarily reflected neither the amount of time passed nor concurrent symptoms, brain patterns may have nonetheless been influenced by individual, unmeasured characteristics. Moreover, the lack of a comparison group complicates the interpretation of observed patterns of findings, particularly as they relate to what we might expect from healthy youth. Thus, our findings are correlational and speak to neural reactivity in response to intervention. Replication in a study designed to include pre-treatment, post-treatment and follow-up fMRI scans could allow for causal inferences to be made. Such a design would also allow for the maintenance of randomization, to better understand the impact of specific treatment paradigms on changes in reward-related circuitry, and vice versa. Future trials that incorporate imaging at multiple time points across multiple treatment arms, including a control condition can use these findings to generate hypotheses to establish directionality of change.

Change in clinical presentation after the receipt of an intervention has implications for participants’ abilities to learn skills within a therapeutic context, and suggests support for treatment match. Findings may also suggest that BBT (and potentially behavioral interventions more broadly) may capitalize on the integration of emotion regulation and social/non-social reward processing networks to promote behavioral activation. Taken together, conclusions should be viewed as preliminary data aimed at hypothesis generation for future work. Ultimately, incorporating behavioral neuroscience tools into clinical science will improve treatment outcomes, as identifying predictors and mechanisms of treatment response is crucial groundwork to move toward a precision medicine approach, including mechanism-based treatment to the individuals whose neural profiles indicate they would benefit the most. This work offers evidence of value in comparing complex data from multiple task contexts and contributes to the establishment of a literature base of neural mechanisms of treatment response in internalizing youth.

## Data Availability

The datasets for this study will not be made publicly available because the data are undergoing secondary analyses in preparation for additional publications.

## Author Contributions

KS, VW, and JW were jointly involved in the study conceptualization and protocol development. KS spearheaded the recruitment for the neuroimaging follow-up, data collection, data preparation, and writing of the manuscript. MK-L led the analytical efforts with ML’s assistance. VW contributed the data from the original RCT and approved the use of the sample for the neuroimaging follow-up. JW provided training to facilitate the study completion, supervised the data collection and analytical efforts, and contributed to the writing of the manuscript. All authors critically reviewed the manuscript and approved it prior to submission.

## Conflict of Interest Statement

The authors declare that the research was conducted in the absence of any commercial or financial relationships that could be construed as a potential conflict of interest.

## Footnotes

1 http://www.macbrain.org/resources.htm

2 https://afni.nimh.nih.gov/afni/
