# Supplementary material for: Social and Non-social Reward: A Preliminary Examination of Clinical Improvement and Neural Reactivity in Adolescents Treated With Behavioral Therapy for Anxiety and Depression
Source: Front Behav Neurosci. 2019 Aug 23;13:177. doi: 10.3389/fnbeh.2019.00177 (PMC6736628; doi:10.3389/fnbeh.2019.00177)

Supplemental Materials

Figure S1. *Face emotion dot probe task.* After a brief presentation of two faces (1 happy, threatening, or sad, 1 neutral; Tottenham et al., 2009), one of the faces is replaced by a probe (“<” or “>”). The participant is instructed to indicate the direction of the probe (right or left) via button press. The probe replaces the emotional face 50% of the time (not shown). An additional trial type (2 neutral faces, not shown) serves as control.

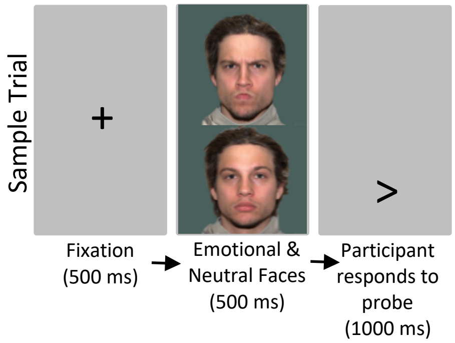

Figure S2. *Child-friendly non-social reward task.* Note: published previously in Wiggins et al. (2017), Journal of Affective Disorders and adapted from Helfinstein (2013), Social Cognitive and Affective Neuroscience.

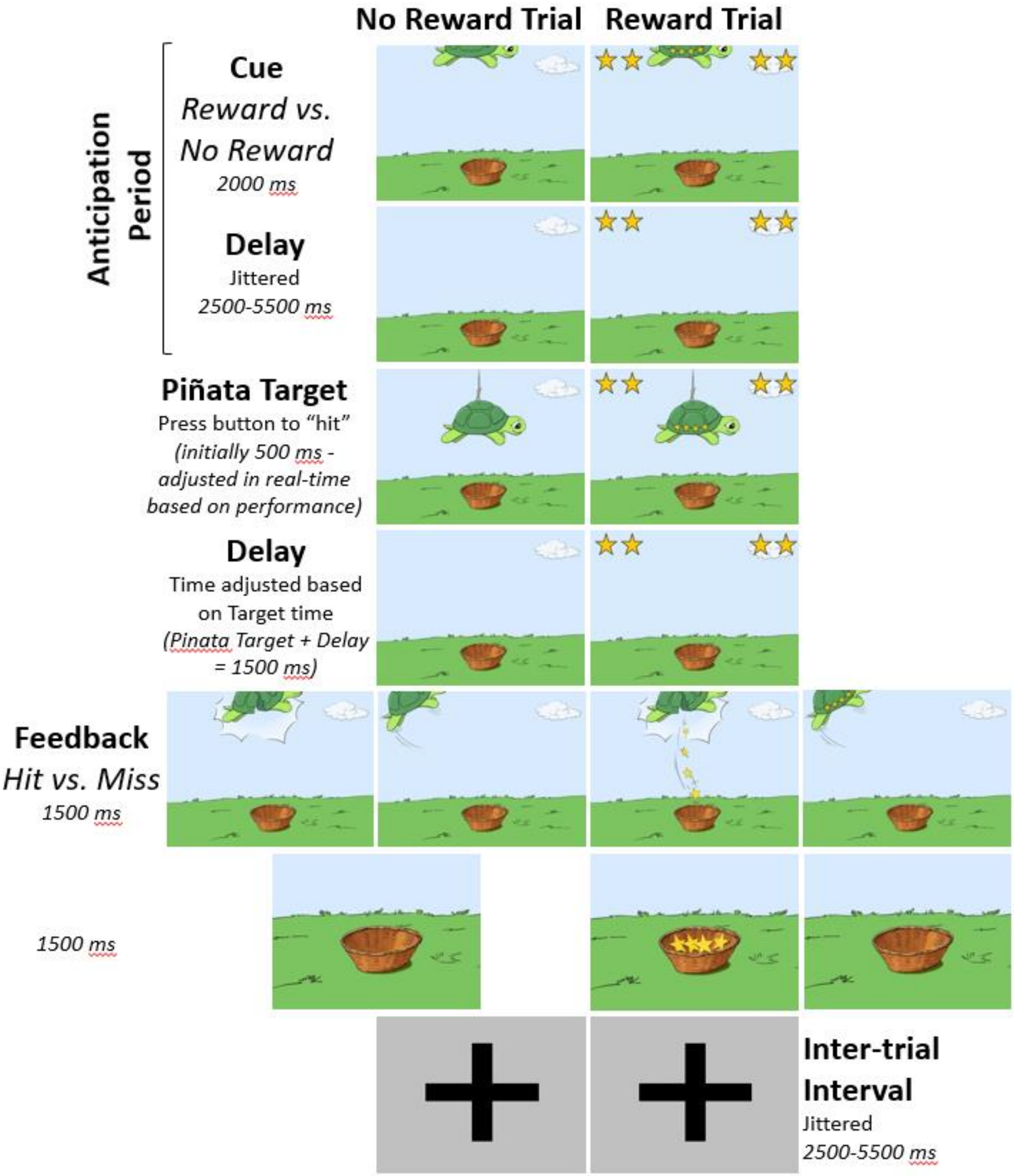

Supplement: Supplementary file 1 [file Data_Sheet_1.pdf]
